# Emergency Medical Services Policies and Perspectives Leading to Ambulance Engine Idling

**DOI:** 10.5811/westjem.47186

**Published:** 2025-09-25

**Authors:** Matthew Lyons, Aaron R. Kuzel, Stephen Marks, Craig Ziegler, Kahra Nix

**Affiliations:** * University of Louisville, School of Medicine, Department of Emergency Medicine, Louisville, Kentucky; † University of Louisville, Office of Graduate Medical Education, Louisville, Kentucky

## Abstract

**Introduction:**

Ambulances are often left to idle, which may contribute to maintenance costs, environmental harm, and resource inefficiencies. Engine idling affects the health of first responders due to the consequences of exhaust. Our study objective was to gain understanding of current emergency medical services (EMS) policies and perspectives on ambulance engine idling.

**Methods:**

We designed an anonymous, 48-question survey that was distributed to all levels of EMS clinicians. There were 684 total survey responses from 11 states. We excluded those that only included demographics, yielding 507 responses. The response rate was 10.8%. The questions surveyed demographics, service characteristics, and current policies and perspectives on idling. We used multiple question types, including some that asked participants to rate their level of concern on a five-point Likert scale. “Strongly disagree” was coded as 1, and “strongly agree” was coded as 5. “Neither agree or disagree” was considered a neutral response and was coded as 3. Additionally, we conducted a thematic analysis on data derived from the free-text responses to identify themes.

**Results:**

Few (12%) respondents reported written policies on idling. The biggest concerns regarding idling involved the following (reported as median (IQR, 25^th^ and 75^th^ percentiles): patient comfort (4, IQR 4–5); EMS clinician comfort (4, IQR 4–5), and medication compromise (4, IQR 4–5). There was a neutral level of concern regarding equipment failure (3, IQR 3–4) and response delays (3, IQR 3–5). There was a less than neutral level of concern regarding engine failure (2, IQR 2–4); vehicle theft (2, IQR 2–4), air quality (2, IQR 2–3); increased fuel usage (2, IQR 2–3); and carbon emissions (2, IQR 2–3). Six themes emerged: fear of harming patient; safety; effects on air quality; habits and indifference; cost of idling; and frustration.

**Conclusion:**

Emergency medical services clinicians mainly hesitate to turn off their engines out of concern for patient/personnel harm and potential equipment failure. The theme of frustration, noted in free-text responses, describes EMS clinicians’ feelings of suspicion and concern for an ulterior motive behind the study, which highlights the need for a collaborative effort at addressing this collective issue.

## INTRODUCTION

Each year, emergency medical services (EMS) clinicians in the US respond to more than 25 million calls.^1^ These calls continue to increase; Medicare beneficiary use of EMS rose by 15% between 2007–2015.^2^ Emergency medical services call responses typically involve one or more ground ambulances powered by internal combustion engines, running on unleaded or diesel fuel. Ambulances are often left to idle while at healthcare facilities and while “available” for service. The vehicles have needs beyond propulsion that require power, including housing equipment, storing medications, and ensuring safe and comfortable compartment temperature. The physical hazard of an EMS clinician’s exposure to weather, which motivates the choice to idle, is a safety concern that goes beyond just comfort.

Engine idling also causes concerns for the health of first responders from exposure to diesel particulate matter.^3^ Diesel exhaust was classified as a human carcinogen (group 1) by the International Agency for Cancer Research in 2012.^4^ The Occupational Safety and Health Administration does not have a standard for diesel exhaust as a single hazard; however, it does have standards for specific components of diesel exhaust such as carbon monoxide, sulfur dioxide, and nuisance dust.^5^ These diesel pollutants can have both short-term (headache, dizziness, and mucosal irritation) and long-term (respiratory disease, cardiovascular disease, cardiopulmonary disease and lung cancer) health consequences.^5^

Research has shown other negative impacts of idling, such as higher maintenance costs. For example, every hour of engine idling may be equivalent to 25 miles of driving.^6^ A study by the US Department of Energy (DOE) found that emergency response vehicles including ambulances routinely idle, and these idling vehicles cost their organization thousands of dollars every year in fuel.^7^ A DOE study that examined use of idling reduction technologies, including auxiliary power units (APU) in emergency response vehicles, found that departments saved an average of $2,600 per year per vehicle.^8^ Additionally, there are environmental and other cost impacts.

No prior studies have investigated the viewpoints of first responders who are fire department based, hospital based, or who work for private and third service-based EMS agencies regarding their attitudes toward engine idling and the preferences and policies motivating this practice. These concerns and the paucity of data about EMS idling practices underscore the need for targeted research. In this study we aimed to obtain quantitative and qualitative data to better understand EMS clinicians’ concerns and perceptions about engine idling.

## METHODS

### Study Design

We distributed an anonymous, 48-question survey using REDCap, a secure, web-based platform (Research Electronic Data Capture hosted at the University of Louisville). There were various question types: five-point Likert scale response items; closed-ended questions; and free-text fields. The questions surveyed the following elements: respondent demographics; vehicle and fuel type; patient population served; and both policies and perspectives around idling. We reviewed the literature and spoke with current EMS clinicians to compile a list of common concerns regarding idling. We ensured the survey’s content validity through literature review and author content expertise (ML, AK, and SM).

We piloted the survey on eligible EMS clinicians who are also content experts, followed by a debrief via cognitive interviewing. We performed additional response-process validity with people outside the EMS community sample, and we consulted a statistician (CZ). The full survey is available as supplementary material. The study was deemed exempt by the University of Louisville Institutional Review Board.

**Population Health Research Capsule**

What do we already know about this issue?

*Engine idling has been previously connected to environmental harm and occupational exposures that affect human health*.

What was the research question?

*What are the current policies and EMS clinician motivations and concerns surrounding ambulance engine idling?*

What was the major finding of the study? *The biggest concerns were patient comfort, clinician comfort, and medication compromise (Median=4, IQR=4, 5)*.

How does this improve population health?

*Expanding policies on engine idling could decrease environmental and occupational harm. However, EMS clinician concerns should be addressed when creating solutions*.

### Participants

Inclusion criteria included active and retired EMS clinicians: emergency medical responders (EMR), emergency medical technicians (basic, advanced, and intermediate), paramedics, and physician EMS medical directors. The survey was distributed by list-servs, social media, and a flyer posted in the emergency department (ED) of the University of Louisville, an urban, academic medical center and tertiary-care center in Louisville, KY. The consent was included on the first screen; thus, completion of the survey acted as implicit consent. The survey was open from July 31–October 5, 2024. There were 684 total responses. Responses that only included demographics were excluded, yielding a total of 507 responses.

Using the American Association for Public Opinion Research standard definition, the response rate for this survey was 10.8%.^9^ Respondents were defined as individuals who potentially received the survey from one of two list-servs or other sources but did not respond. It is not known whether these individuals did not respond because they were ineligible or because they were eligible but chose not to respond. We took a conservative approach when calculating the response rate, and all these individuals were included in the denominator. It is likely that the actual response rate was higher, as it is unlikely that every individual was eligible.

### Statistical Methods

**Figure 1:**
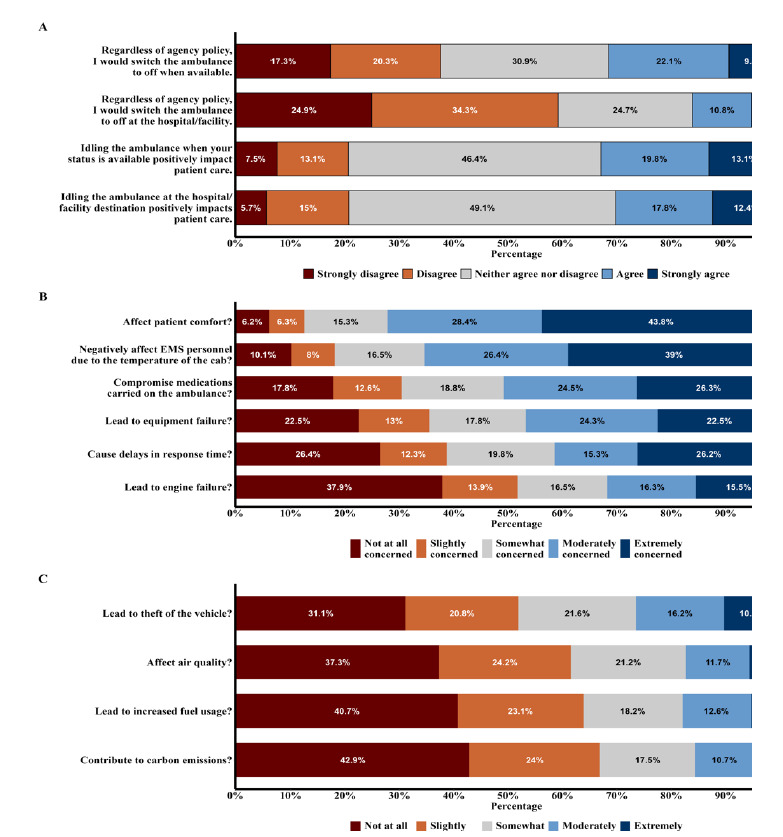
Emergency medical services clinicians’ responses regarding their level of concern about policies and common issues surrounding ambulance engine idling.

We calculated simple descriptive statistics for all demographic and auxiliary variables. For continuous variables, we calculated means and SDs or medians and IQR (25^th^ and 75^th^ percentiles) depending on the normality of the distributions. For nominal variables, frequencies and percentages were calculated. For data analysis, we converted the five-point Likert response format items to numerical values. “Strongly disagree” was coded as 1 and “strongly agree” as 5. “Neither agree or disagree” was considered a neutral response and was coded as 3. Medians of these items are reported in the text, while percentages are depicted in Figure 1.

In Figure 1, Stratum A illustrates responses about current preferences of respondents regarding ambulance engine idling, considering agency policy and whether respondents believe ambulance engine idling affects patient care. Stratum B illustrates potential concerns regarding not idling the ambulance engine. Stratum C illustrates potential concerns regarding idling the ambulance engine. Data values are based on a Likert scale from 1 (not at all concerned/strongly disagree) to 5 (extremely concerned/strongly agree).

We used a Kruskal-Wallis non-parametric test to assess the difference among levels of training and practice settings on the items in Likert response format. For practice setting, when an item was statistically significant, we performed pairwise comparisons between the groups using a Dunn post hoc test with a Bonferroni correction to adjust for possible inflated type I error rates. For levels of training, using the Dunn-Bonferroni correction showed no significant pairwise comparisons with a Bonferroni correction to adjust for the fact that testing multiple outcomes inflated the experiment-wise type I error rate. Key findings are reported in the text as medians.

We used a Spearman correlation coefficient to assess the association between length of service with the Likert response format items. When the Benjamini Hochberg test was performed to minimize the risk of type 1 error rates, only one correlation was significant; thus, we report non-adjusted *P*-values due to the risk of making a type 2 error. All *P*-values were two-tailed. Statistical significance was set by convention at *P* < .05. We used SPSS v29.0 (SPSS Statistics, IBM Corp, Armonk, NY) to analyze the data, and we used the R Package ggplot2 (The R Foundation for Statistical Computing, Vienna, Austria) to produce Figure 1.

There were seven free-text prompts. Two allowed clarifications of existing written idling policies when the ambulance was at a facility destination and while it was “available” for service. Two prompts each allowed participants to clarify why they felt idling at the facility destination or while “available” for service positively affected patient care as a follow-up to two Likert-scale questions. One prompt followed Likert-scale questions to allow clarification of any additional concerns regarding idling. Participants also had a free-text response option to share their personal opinions regarding idling in general while at the facility destination and while “available” for service. To identify and interpret the themes in the qu-alitative dataset, we conducted a systematic thematic analysis through the six steps described by Naeem.^10^ ML performed the initial review, coding of responses, derivation of themes, and conceptualization. AK, SM, and KN reviewed and verified the selection of keywords, coding of the data, development of themes, and conceptualization.

## RESULTS

### Descriptive Data

Demographic characteristics are reported in Table 1. Respondents varied in level of training, years of service, practice setting, and shift duration. Participants characterized their current or previous service. These are reported in Table 2.

### Quantitative Analyses

Respondents had a neutral response when asked whether idling positively impacted patient care either while the ambulance was “available” (Median 3) or at the hospital/facility destination (Median 3). When asked if given the option, regardless of agency policy, respondents had an overall neutral opinion on turning the engine off both when “available” (Median 3) and at the hospital/facility destination (Median 2). Respondents were asked how concerned they were about various impacts of idling, and the results are illustrated in Figure 1. The most prevalent concerns leading to the decision to idle were patient comfort and clinician comfort. Results showed a slightly greater than neutral concern regarding medication compromise, equipment failure, and delays in response time. Responses declared a slightly less than neutral concern for engine failure, theft of vehicle, air quality, increased fuel usage, and contribution to carbon emissions.

To identify confounders, concerns were differentiated based on level of training, practice setting, and length of service. Differences in median level of concern among different levels of training are represented in Table 3. Differences in median level of concern among EMS clinicians in urban, suburban, and rural practice settings are represented in Table 4.

There were statistically significant correlations between EMS clinicans’ length of service with their concern for patient comfort (*R*_s_ = .108, *P* =.02); compromise of medications (*R*_s_ =.153, *P* < .001); and increased fuel usage (*R*_s_ = .097, *P* = .03).

### Qualitative Analyses

We performed a qualitative analysis to identify concerns that were not anticipated in the survey during study design. Nine codes were derived: fear of equipment failure; temperature; security; time at facility; unit readiness; air quality; economics; indifference; and frustration. The most common code was “fear of equipment failure” (*n* = 167), followed by “temperature” (*n* = 87). There were six themes derived: fear of harming patients; perceived level of safety; effects on air quality based on ambulance bay design and age of ambulance; idling due to previously established habits and indifference; perceived cost of idling; and frustration. Representative quotes are presented in Table 5.

#### Theme 1: Fear of Harming Patients

This was the most prevalent theme in the analysis and encompassed a range of concerns that EMS clinicians felt would ultimately impact patient safety. Respondents cited degradation of medications outside acceptable temperature ranges as a common concern. Additionally, EMS clinicians were concerned that turning off the engine would cause equipment or engine failure; they were also concerned that this could cause a delay in response time or an inability to even respond to calls. This likely reflects how operational readiness is a core EMS value.

#### Theme 2: Perceived Level of Safety

This highlighted a reason that some EMS clinicians choose not to idle. It was noted by some to be an especially important consideration in rural areas. However, it was mentioned that newer ambulances have anti-theft devices that are active with the engine idling.

#### Theme 3: Effects on Air Quality Based on Ambulance Bay Design and Age of Ambulance

Some respondents shared concern about the consequences that idling has on air quality. They identified poorly designed ambulance bays that did not include appropriate ventilation or emission controls. Concerns were directed toward ambulance bay design and age of the ambulance more than the act of engine idling. Respondents were not as concerned about emissions from newer trucks, but they did acknowledge that older ambulances might contribute more to poor air quality.

#### Theme 4: Engine Idling Due to Previously Established Habits and Indifference

Respondents stated that they idle simply because it is what they have historically done. Additionally, there were EMS clinicians who reported indifference to whether there could be impacts from idling suggesting that idling may be a habit, rather than a deliberate choice informed by operational or environmental needs.

#### Theme 5: Perceived Cost of Engine Idling the Ambulance

Administrative or supervisory personnel are more likely to highlight the potential costs of idling. These respondents emphasized cumulative costs across an entire department fleet. This highlights the role that improved efficiency may play in operational and budgetary challenges faced by EMS organizations.

#### Theme 6: Frustration

While there was constructive feedback, there were frustrations as well. Some respondents highlighted that the study was being conducted out of the ED rather than an EMS service, which led to a feeling of suspicion or concern for an ulterior motive regarding the study objectives. Others believed that the hospital or other public service agencies should be the focus when addressing the negative consequences of idling.

## DISCUSSION

Decreasing idling would lead to health, cost, and environmental benefits. In this survey of EMS clinicians, we explored the current policies and perspectives regarding engine idling. Only 12% of respondents reported that their agency has a written policy regarding idling, suggesting that the decision to idle is predominantly the choice of the individual responder. Open-ended responses in this study indicated that EMS clinicians are open to decreasing idling if these potential harms are addressed. Following are the foremost areas of concern identified in the thematic analysis and should each be considered when approaching solutions.

### Patient Comfort

Patient comfort was a chief concern that EMS clinicians of all levels reported when asked about switching off the engine. Of the respondents, 72% indicated that they were either moderately or extremely concerned that not idling could affect patient comfort. Medical directors had a significantly lower level of concern for patient comfort compared to all other types of EMS clinicians. There are national standards for the minimum and maximum temperatures that the cab and chassis must meet when manufactured.^11^ However, operating temperature standards are dependent on whether local policy exists. Washington, DC, is one area that has a written policy requiring that the patient compartment be between 50°F-85°F while awaiting a patient response.^12^ Aside from patient comfort, poorly regulated temperature in the patient compartment can also have effects on patient outcomes. Ambulances have a limited ability to quickly achieve and maintain these temperatures, particularly in winter months.^4^, ^13^

### Emergency Medical Services Clinician Comfort

Emergency medical services clinician comfort, although less concerning to EMS clinicians than patient comfort, was a worry for respondents. In this study 61% of respondents reported working shifts longer than 24 hours. The amount of time spent in the ambulance varies based on several factors, including service structure, run volume, dynamic or fixed deployment models, and the size of the service area. It is not uncommon for some to be in the ambulance nearly the entirety of their shift. High levels of EMS clinician burnout are well documented in the literature, with one study reporting rates of nearly 60% across services in North Carolina.^14^ Regardless of burnout or the current nationwide EMS clinician shortage, comfort should be prioritized. Agencies should consider clinician access to a temperature-controlled area or an alternative system, like APUs, to control the temperature of the cab if the engine is to be switched off while occupied.

### Medication Storage

Many respondents cited a concern about medication safety and quality in the absence of idling. Most medications on an ambulance are regulated by the United States Pharmacopeia (USP) to be stored at “controlled room temperature,” which is defined as approximately 59–86°F.^15^ In one observational study, it was found that across five different EMS services in different climate regions, no ambulance was able to consistently maintain a medication storage temperature within USP recommendations.^13^ This is likely exacerbated by switching the engine off. An APU or shore power would need to be employed to mitigate this issue when not idling.

### Equipment and Engine Failure

Some ambulance components are dependent on a power supply. With the modernization of ambulances, such as the creation of the power stretcher, a reliable power supply is crucial for operational preparation. Forty-seven percent of respondents were moderately or extremely concerned that not idling could lead to equipment failure. The thematic analysis provided more context for this concern. “Fear of equipment failure” was the most common code in the thematic analysis. Review of free-text responses revealed that many EMS clinicians reported having to provide patient care in ambulances that are dated and poorly maintained. Recently, the US Centers for Disease Control and Prevention recognized that EMS services nationwide are “severely underfunded.”^17^ This in turn leads to a limited budget for maintenance, expansion, and modernization of the fleet.

There was discordance between some free-text responses and the Likert-scale responses regarding the concern for potential engine failure. Some respondents reported instances of an engine that would not restart after being turned off, which has obvious and notable implications for a service’s ability to provide timely emergency care. Respondents pointed out that this was particularly an issue in rural services where there are very few ambulances in service and patients are often transported longer distances for higher levels of care.

### Delays in Response Time

The thematic analysis revealed that the concern for delays in response time was predominantly within the rural community. This concern was separate from the worry that the engine would not restart, and the motivator for this specific concern is unclear. Based on responses, this concern seems to be that the time required to start the engine could lead to a delay. There is no current literature to clarify this.

### Theft of the Vehicle

Idling an ambulance leaves it vulnerable to theft and crimes of convenience. Twenty-six percent of respondents were moderately or severely concerned about idling leading to theft of the vehicle, and rural EMS clinicians were significantly more concerned compared to others. A review from 2020 showed that ambulance thefts are increasing nationwide.^18^ There are devices commercially available that allow the key to be removed from the ignition while the vehicle is running, which would allow the doors to lock while idling.^19^ These devices address the issue of theft, but they do not provide the cost and environmental benefits of turning off the vehicle. Additionally, this could be limited in older ambulance models.

### Air Quality and Emissions

Respondents reported, on average, that the engine is on for approximately 58% of their shift. The most commonly reported shift was 24 hours. It is well documented that idling has negative effects on the environment. Idling causes emission of harmful combustion products, including carbon monoxide, nitrous oxides, and other greenhouse gases.^20^ As mentioned by some respondents, the presence of these byproducts can be especially taxing in those who have respiratory diseases and are exposed while in the ambulance bay or ED entry. In this study 66.7% of respondents reported that their service’s vehicles primarily use diesel. Diesel engines, on average, emit 1.14 times the amount of carbon dioxide as compared to gasoline engines. Some respondents argued that filters can mitigate this discrepancy. A diesel particulate filter (DPF) is a modification to a diesel vehicle that can significantly reduce particulate matter, nitrous oxide, and carbon monoxide.^21^ Our survey did not ask respondents about the presence or absence of a DPF on their vehicles, and their prevalence on ambulances is unknown. Suburban EMS clinicians were significantly more concerned about air quality than rural EMS clinicians, although both had a low concern overall.

### Increased Fuel Cost

Physician respondents were significantly more concerned about increased fuel usage due to engine idling compared to the others. Urban EMS clinicians were significantly less concerned about fuel usage than other EMS areas. A case study by Argonne National Laboratory found that with battery APUs, ambulance services saved at least $5,100 in annual fuel cost per ambulance.^22^ There is significant variability between APUs with varying cost savings.^23^ Given the general underfunding of EMS systems in the United States, reducing idling presents a potential area to decrease costs.

### Potential Solutions

The issues surrounding idling are complex. We identified numerous variables that are concerns for EMS clinicians and deserve recognition when trying to address the issue:

The first APU, a device that provides energy for functions other than propulsion, was designed for aviation use in the 1950s.^25^ Since then, usage has been expanded to fire and police vehicles, and ambulances. Generally, they can provide power to certain components of the ambulance when the engine is off. There is significant variability between APUs, including the number of components it can power and how long it can run with the engine off. In addition to showing an annual cost savings of $5,100, lead-powered APUs reduced oil changes by at least 50%.^23^ Based on their calculations, the APUs would recuperate their cost in one to two years, making them an economical option for EMS services. In this study, 41.9% of respondents reported having an APU that would allow access to power while the engine is off. The frequency of APU use was not investigated in this study.

Electrified parking spaces (EPS) are another potential solution that would provide similar benefits to APUs. These are parking spaces that provide a shoreline or duct system to augment power while the engine is off. A study of one commercial EPS unit was shown to cost $0.75 per hour to operate, compared to an estimated $18.75 per hour to operate an ambulance that is running.^4^ One anticipated barrier to the use of EPS is that installation at hospitals would increase the facility’s electricity costs. Because of this, non-hospital-based EMS services may have difficulty with hospital buy-in on installation despite the promise of cost savings and lessening environmental and health effects. Furthermore, dynamic deployment models of EMS may preclude the ability of ambulances to connect to EPS.

It should be acknowledged that throughput time at healthcare-facility destinations is a notable contributor to idling. Compounding this is poor ambulance bay design, which limits ambulance movement or does not provide appropriate parking areas to allow an engine to be switched off. Improving these are potential solutions as well.

## LIMITATIONS

We attempted purposive sampling; however, there were responses from only 11 US states with 92.6% of respondents working in Kentucky. The response rate was only 10.8%, and there was also greater representation of self-identified White (96.7%), males (75.6%), and paramedics (47.5%). However, a national survey of EMS clinicians with 87,471 respondents from 46 states found 82.4% to be White, 75.8% to be male, and 44.9% to be paramedics, indicating this greater representation closely mirrors the national EMS workforce.^26^ Clearly, a response rate of 10.8% leads to a rational concern that the responses may be biased. However, in most surveys, when bias occurs, it is typically those at the extremes of opinions and not the less extreme responses. In examining our data, the modal response to our survey in Stratum “A” in Figure 1 were the most neutral responses for three of the four questions posed. This suggests bias may be minimal. On the other hand, the modal responses for Stratum “C” were “not at all concerned” for all four questions in this stratum, and this could reflect bias. Thus, we believe we are receiving “mixed signals” regarding whether our low survey-response rate was a source of bias.

The survey was distributed during only one weather season, which could be a potential limitation to generalizability. Local EMS clinicians shared with us a fear of consequences due to the potential that their personal practices or opinions might conflict with their EMS agency, which could have introduced bias. We attempted to minimize this by making all questions optional and adding a preamble reminding respondents that the survey was anonymous and data would not be shared directly with employers. However, this could have added bias in allowing respondents to selectively answer questions. There was potential for recall bias when respondents were asked to describe written policy regarding idling. (Likert-scale survey questions were shuffled to avoid question-order bias.) Additionally, as with all surveys there was a potential for self-selection bias.

Initially, during the data analysis phase, we applied post hoc corrections to account for multiple testing and the risk of inflating type I errors rates. However, for some series of tests such as with levels of training, length of service, and one item within practice setting (“contribute to carbon emissions”), the corrections led to no significant results that could potentially have increased type II errors. Therefore, we report no post hoc corrections for these variables with the caveat that some caution should be taken with interpretation of the results.

## CONCLUSION

Few survey respondents (12%) reported written policies on idling. Respondents recognized the potential benefits of decreasing idling, including lower fuel costs, fewer negative health effects and emissions, and a lesser likelihood of vehicle theft. However, responses suggested a hesitation to turn off engines due to concern for harm (patient and clinician) and potential engine or equipment failure. Each of these concerns should be considered and addressed when approaching solutions to idling.

Next steps include clarifying that solutions entail far more than issues of fuel cost or wear on the ambulance; exhaust exposure is a documented harm to EMS clinicians. As the thematic analysis revealed frustrations by EMS clinicians (evidenced by feelings of suspicion and concern for ulterior motives by study investigators), it is imperative that solutions be collaborative and efforts reflect that this is a collective issue that cannot be solved by EMS agencies alone.

## Figures and Tables

**Table 1:** Demographics of emergency medical services clinicians who participated in a survey regarding existing policies and their perspective on ambulance engine idling.

What is your current role? *n*, %	Paramedic	240	(47.5%)
	Emergency medical technician (EMT)	193	(38.2%)
	Advanced EMT	54	(10.7%)
	Physician	6	(1.2%)
	Retired	5	(1.0%)
	Emergency medical responder (EMR)	1	(0.2%)
	EMT intermediate	0	(0.0%)
	Other	6	(1.2%)
	No response	2	
Years of service, median (IQR)		13 (5–22)	
Sex (*n*, %)	Male	381	(75.6%)
	Female	121	(24.0%)
	Non-binary	2	(0.4%)
	Other	0	(0.0%)
	No response	3	
Which of the following best describes your race? *n*, %	White	473	(96.7%)
	Black	6	(1.2%)
	Asian	1	(0.2%)
	Other	9	(1.8%)
	No response	18	
Which of the following best describes your ethnicity? *n*, %	Hispanic	7	(1.4)
	Non-Hispanic	483	(98.6%)
	No response	17	
Age, mean (SD)		39.3 (11.9)	

IQR, interquartile range; SD, standard deviation.

**Table 2:** Practice setting and service characteristics of emergency medical service’s clinicians represented in the survey.

Which of the following best describes your service? *n*, %	911 service	460	(91.1%)
	Hospital-affiliated transport	28	(5.5%)
	Private transport	17	(3.4%)
	No response	2	
Which of the following best describes your service? *n*, %	Rural	202	(40.2%)
	Suburban	166	(33.0%)
	Urban	135	(26.8%)
	No response	4	
Which of the following best describes your service? *n*, %	Third-service (operated independently of a fire department)	194	(38.9%)
	Fire-based (operated within the fire department)	147	(29.5%)
	Private company	99	(19.8%)
	Hospital-affiliated	59	(11.8%)
	No response	8	
Does your service offer Advanced Life Support services, or Basic Life Support services only? *n*, %	Advanced Life support	486	(97.2%)
	Only Basic Life Support	14	(2.8%)
	No response	7	
Does your ambulance have an auxiliary power unit to enable you to switch your engine off and still have access to power? *n*, %	Yes	211	(41.9%)
	No	244	(48.4%)
	I don’t know	49	(9.7%)
	No response	3	
Which type of fuel does your services vehicles primarily use? *n*, %	Diesel	337	(66.7%)
	Unleaded	168	(33.3%)
	Electric	0	(0.0%)
Which of the following best describes your patient population? *n*, %	Adult/pediatric mixed	434	(86.3%)
	Adult specialty	66	(13.1%)
	Pediatric specialty	3	(0.6%)
	No response	4	
How long in duration are your shifts? *n*, %	8 hours	3	(0.6%)
	12 hours	134	(26.5%)
	16 hours	43	(8.5%)
	24 hours	299	(59.2%)
	48 hours	11	(2.2%)
	Other	15	(3.0%)
	No response	2	

**Table 3A:** Emergency medical service clinicians’ concerns regarding the potential effects of NOT idling the ambulance engine, on a Likert scale from “not at all concerned” (1) to “extremely concerned” (5). Medians and IQRs are presented.

	EMR (*n* = 1)	EMT (n = 193)	Advanced EMT (*n* = 54)	Paramedic (*n* = 240)	Physician (*n* = 6)	Retired (*n* = 5)	Other (*n* = 6)	P =

	A	B	C	D	E	F	G	
Affect patient comfort?	2 (2, 2)	4 (3, 5) ^E^	4 (3, 5) ^E^	4 (3, 5) ^E^	2 (1, 4) ^B^, ^C^, ^D^, ^F^, ^G^	5 (5, 5) ^E^	5 (4, 5) ^E^	.05
Negatively affect EMS personnel due to the temperature of the cab?	3 (3, 3)	4 (3, 5)	4 (3, 5)	4 (3, 5)	3 (1, 3)	5 (4, 5)	4 (3, 5)	.31
Compromise medications carried on the ambulance?	1 (1, 1)	3 (2, 4)	4 (2, 4)	4 (2, 5)	1 (1, 3)	4 (2, 5)	4 (2, 5)	.12
Lead to equipment failure?	1 (1, 1)	3 (2, 4)	3 (2, 5)	3 (2, 4)	1 (1, 2)	4 (3, 5)	4 (1, 4)	.21
Cause delays in response time?	1 (1, 1)	3 (2, 5) ^E^, ^F^	3 (2, 4) ^E^, ^F^	3 (1, 4) ^E^, ^F^	1 (1, 1) ^B^, ^C^, ^D^, ^F^, ^G^	5 (4, 5) ^B^, ^C^, ^D^, ^E^	4 (1, 5) ^E^	.02
Lead to engine failure?	1 (1, 1)	2 (1, 4)	2 (1, 4)	2 (1, 4)	1 (1, 1)	2 (1, 5)	5 (1, 5)	.19

Superscript lettering in a cell indicates where significant pairwise comparisons between roles are found. For example, in looking at the item “cause delays in response time,” the overall significance of .02 indicates that somewhere among the different levels of training there is a significant difference. The role “physician” is designated [E], and the superscript lettering that shows the ratings were significantly different from EMTs [B], advanced EMTs [C], paramedics [D], retired personnel [F], and other [G]. We used the Dunn test to assess post hoc pairwise comparisons.

*EMR*, emergency medical responder; *EMT*, emergency medical technician; *EMS*, emergency medical services.

**Table 3B:** Emergency medical service clinicians’ concerns regarding the potential effects of idling the ambulance engine.

	EMR (*N* = 1)	EMT (*N* = 193)	Advanced EMT (*N* = 54)	Paramedic (*N* = 240)	Physician (*N* = 6)	Retired (N=5)	Other (N=6)	P =

	A	B	C	D	E	F	G	
Lead to theft of the vehicle?	4 (4, 4)	3 (1, 4)	2 (1, 3)	2 (1, 3)	3 (2, 3)	3 (3, 4)	3 (2, 4)	.69
Affect air quality?	1 (1, 1)	2 (1, 3)	2 (1, 2)	2 (1, 3)	3 (1, 4)	1 (1, 2)	3 (2, 3)	.29
Lead to increased fuel usage?	4 (4, 4)	2 (1, 3) ^E^	2 (1, 3) ^E^	2 (1, 3) ^E^	4 (4, 4) ^B^, ^C^, ^D^, ^G^	2 (2, 4)	2 (1, 2) ^E^	.04
Contribute to carbon emissions?	2 (2, 2)	2 (1, 3) ^C^, ^D^	1 (1, 2) ^B^	2 (1, 3) ^B^	3 (1, 4)	1 (1, 2)	2 (2, 3)	.01

The significance level is based on the Kruskal-Wallis test. Because only one respondent was an EMR, that data was not included in the analysis.

*EMR*, emergency medical responder; *EMT*, emergency medical technician; *EMS*, emergency medical service.

**Table 4A:** Emergency medical service clinicians’ concern regarding the potential effects of NOT idling the ambulance engine, based on their service location.

	Urban (*n* = 135)	Suburban (*n* = 166)	Rural (*n* = 202)	P =

	A	B	C	
Affect patient comfort?	4 (4, 5)	4 (3, 5)	4 (3, 5)	.41
Negatively affect EMS personnel due to the temperature of the cab?	4 (4, 5) ^B^, ^C^	4 (3, 5) ^A^	4 (3, 5) ^A^	.001
Compromise medications carried on the ambulance?	4 (2, 5)	3 (2, 4)	4 (2, 5)	.70
Lead to equipment failure?	4 (2, 4)	3 (2, 4)	3 (2, 4)	.62
Cause delays in response time?	4 (2, 5) ^B^	3 (1, 4) ^A^	3 (1, 5)	.01
Lead to engine failure?	2 (1, 4)	2 (1, 4)	3 (1, 4)	0.42

*EMS*, emergency medical services.

**Table 4B:** Additional concerns reported by emergency medical service clinicians regarding the potential effects of idling the ambulance engine, based on their service location.

	Urban (*n* = 135)	Suburban (*n* = 166)	Rural (*n* = 202)	P =

	A	B	C	
Lead to theft of the vehicle?	2 (1, 3) ^C^	2 (1, 3)	3 (2, 4) ^A^	.001
Affect air quality?	2 (1, 3) ^B^	2 (1, 3) ^A^, ^C^	2 (1, 3) ^B^	.03
Lead to increased fuel usage?	1 (1, 3) ^B^, ^C^	2 (1, 3) ^A^	2 (1, 3) ^A^	.002
Contribute to carbon emissions?	2 (1, 3) ^B^	2 (1, 3) ^A^, ^C^	2 (1, 3) ^B^	.04

The significance level is based on the Kruskal-Wallis test. Superscript lettering in a cell indicates where significant pairwise comparisons between roles are found. For example, by looking at the item “contribute to carbon emissions,” the overall significance of .04 indicates that somewhere among the different practice settings there is a significant difference. The location suburban is lettered [B], and the lettering shows the ratings are significantly different from urban [A] and rural [C]. We used the Dunn test with Bonferroni correction to assess post hoc pairwise comparisons. For the item “contribute to carbon emissions,” no significant pairwise differences were found between locations when the Bonferroni correction was used, so only the Dunn test (without a correction) is depicted.

*EMS*, emergency medical service.

**Table 5:** Representative quotes used to derive themes from a survey of emergency medical service clinicians regarding their perspectives surrounding ambulance engine idling.

**Theme 1** – Fear of harming patients	“Idling allows for the truck to stay at an appropriate temperature, protect equipment and medications, and prevents the truck from not starting when leaving the hospital en route to another call.” “We have a lot of things in the ambulance that operate on battery including internet, GPS, MDT system, stretcher, etc. All of these things can drain the batteries relatively quickly, and if the ambulance’s battery is dead we cannot respond to the next emergency.” “I would rather be idling in case the engine is off and would not start and cause a delay in transport.”
**Theme 2** – Perceived level of safety	“Ambulances can be and should be equipped with a safety feature that allows engine to idle with key out. Our ambulances allow you to idle the engine with the key out and vehicle secured/locked. If someone was to get in and touches the break, the engine will stop preventing theft.” “I do worry about it being stolen, but I also worry about it not restarting.” “I believe that ambulances should not be left idling in the ambulance bays. It’s led to multiple vehicle thefts in the county.”
**Theme 3** – Effects on air quality based on ambulance bay design and age of ambulance	“The design of the ambulance bay is up to the hospital administration. Most facilities I’ve encountered have fans or ventilation systems at the EMS entrance to their ED as well as two sets of doors that prevent exhaust fumes from entering the facility.” “Turned off only if the air filtration system draws the exhaust fumes into the ER or hospital.”
**Theme 4** – Engine idling due to previously established habits and indifference	“I could care less.” “Frankly, I don’t think about these things.” “It’s mostly idled due to habit. I’ve never been instructed by policy to keep it running but always have.”
**Theme 5** – Perceived cost of engine idling the ambulance	“I believe that turning the unit off is more economically sound from a business perspective, however hospitals are not set up or equipped to dock/charge units that are turned off. “ “Waste of gas and higher theft chances.”
**Theme 6** – Frustration	“Hospital/facility destinations should not dictate the policies and procedures of the separate entities that own and operate ambulances.” “Why are we so focused on ambulances, seems kinda like a vendetta.”

*GPS*, global positioning system; *MDT*, mobile data terminal; *EMS*, emergency medical services; *ED*, emergency department; *ER*, emergency room.
